# Recent Advances and the Mechanism of Astaxanthin in Ophthalmological Diseases

**DOI:** 10.1155/2022/8071406

**Published:** 2022-05-20

**Authors:** Ming Yang, Yanling Wang

**Affiliations:** Department of Ophthalmology, Beijing Friendship Hospital, Capital Medical University, No. 95 Yong'an Road, Xicheng District, Beijing 100050, China

## Abstract

Astaxanthin (AST) is a naturally occurring carotenoid that has strong antioxidant, anti-inflammatory, and antiapoptosis effects and is used for the prevention of cancer. There is growing evidence that AST has multiple protective effects against various eye diseases. This article reviews the function and the potential mechanism of AST in dry eye syndrome, keratitis, cataract, diabetic retinopathy, age-related macular degeneration, high intraocular pressure, and other ocular diseases. It provides a theoretical basis for the clinical application of AST as a potential nutraceutical.

## 1. Introduction

Astaxanthin (AST) is a lipid-soluble and red-orange carotenoid pigment that belongs to a group of carotenoids called xanthophylls [1, 2]. In 1938, Kuhn et al. originally discovered AST in lobsters as a source of pigment in aquaculture. It was found in various microorganisms and marine organisms, such as yeast, microalgae, salmon, and krill, as well as plants, such as the leaves, flowers, fruits, and feathers, playing a role mainly in color rendering [3–5]. In 1991, AST was approved as a food supplement, and its biological activity, antioxidant properties, and physiological properties as a precursor to vitamin A were reported in rats and fish [6]. The chemical name of AST is 3,3′-dihydroxy-4,4′-diketo-beta, beta′-carotene [7]. It has an extended structure with a polar region at both ends of the ion ring, which gives it the ability to neutralize free radicals. There is also a nonpolar zone in the middle, consisting of a series of carbon-carbon double bonds called “conjugation.” Compared with 11 *β*-carotene, AST with 13 conjugated double polyunsaturated bonds has unique chemical properties, molecular structure, and light absorption properties [8]. The hydroxyl group at the 3,3′*β* position and the ketone portion of each ionone ring make the AST molecule more polar, greatly enhancing its membrane function. The polar-nonpolar-polar structure of the AST molecule allows it to precisely match the polar-nonpolar-polar regions of the cell membrane [9]. In comparison to other carotenes, AST has several common physiological and metabolic activities.

Due to the specific biological structure, AST can play an important role in a variety of biological processes, such as antioxidant, anti-inflammatory, antiapoptotic, and anticancer processes; it has a series of biological functions, such as protecting the cardiovascular system, delaying diabetic nephropathy and liver injury, having antiobesity properties, and improving immunity [10]. Currently, AST has become a hot research topic, and several recent studies have highlighted the potential protective role of AST in eye diseases. Although AST is not present in the retina, such as lutein and zeaxanthin, it can selectively cross the BRB and protect the retinal cells from oxidative damage [11]. Moreover, the prognosis of various eye diseases has significantly improved, including that of ocular surface diseases (dry eye and keratitis), fundus diseases (diabetic retinopathy, age-related macular degeneration, and ischemia), cataracts, and optic nerve-related diseases [11–17].

In our review, we systematically summarized and highlighted the protective effects of AST on eye diseases. The effects of AST on ocular tissue cells and its molecular mechanisms involved, such as the regulation of antioxidant and antioxidant activation, the regulation of immune response, the prevention of cell damage, and the regulation of apoptosis, were discussed further. This review article discussed the therapeutic potentials and health benefits of AST in ocular disease.

## 2. Main Biological Activities of AST

### 2.1. Antioxidant Activity

Oxidative stress is caused by the imbalance of oxidation or the antioxidant reaction in cells, which promotes the production of excessive reactive oxygen species and free radicals [18]. Excessive oxidative factors are an important mediator in the pathological process of diseases. They may react with proteins, lipids, and DNA through chain reaction to induce protein inactivation, lipid oxidation, and DNA damage, causing many diseases [19]. The structure of AST determines its strong antioxidant ability. The polyene chain can capture free radicals in the cell membrane, and the terminal ring can scavenge free radicals inside and outside the cell membrane [20]. Therefore, AST protects the membrane structure by neutralizing singlet oxygen, preventing chain reactions, and inhibiting lipid peroxidation (LPO), hence, increasing the function of the antioxidant enzymes [21, 22]. AST can increase the levels of antioxidants catalase (CAT), superoxide dismutase (SOD), and glutathione peroxidase (GPx) in the hippocampus of type 2 diabetic rats, therefore, increasing the antioxidant capacity of the hippocampus [23]. In addition, another survey showed that AST can significantly increase the activity of GPx and SOD, protecting mouse hepatocytes from oxidative damage by using hydrogen peroxide [24]. Fakhri et al. also found that AST can increase the expression of glutathione S-transferase *α*1 (GST-*α*1), which enhances the biological defense against oxidative stress damage via the activation of the extracellular signal-regulated protein kinase (ERK) signaling pathway in vitro and in vivo [10].

### 2.2. Anti-Inflammatory Activity

Inflammation is a complex series of immune responses, which act as a host defense mechanism or a response to body damage when initiating tissue repair process [25]. However, excessive or uncontrolled inflammation is harmful to the host and may cause damage to the host cells and tissues [26]. AST is a strong antioxidant, which can prevent inflammation of the biological system. It plays an important role in preventing the progression of central nervous system diseases and protecting both acute and chronic organs (lung, kidney, and liver) inflammatory injury. Further researches showed that AST blocks the nuclear factor kappa-B (NF-*κ*B) dependent signaling pathway; it also forestalls gene expression of downstream inflammatory mediators, such as interleukin-1*β* (IL-1*β*), interleukin-6 (IL-6), and tumor necrosis factor-*α* (TNF-*α*) [22, 27–29]. It suppresses the levels of inflammatory factors (TNF-*α* and IL-6) through the inhibition of mitogen activated protein kinase (MAPK) and nuclear factor-activated pathways, which prevents acute lung injury [30]. In addition, AST can reduce the expression of interleukin-8 in infected gastric epithelial cells [31]. Moreover, Han et al. found that AST prevents ethanol-induced hepatic injury via directly binding to the DNA binding site of STAT3 and inhibiting its activity; this reduces inflammatory responses [32].

### 2.3. Antiapoptosis Activity

Recent studies have shown that apoptosis is associated with many diseases, such as autoimmune diseases, neurodegenerative diseases, tumors, AIDS, and multiple organ dysfunction syndrome [33–36]. AST can modify some key apoptotic proteins to prevent the occurrence of related diseases [37]. Kowshik et al. reported that AST can promote cell apoptosis in oral squamous cell carcinoma models, and it plays a role as a tumor suppressor by downregulating noncoding RNAs, miR-21, and HOTAIR via the PI3K/Akt pathway [38]. AST can induce antiapoptosis activity in the classic mitochondrial pathway, which is closely related to reactive oxygen species (ROS), Bax, Bcl-2, P53, and other molecules. For example, AST can protect renal tubular epithelial cells from acute injury by downregulating proapoptotic proteins, P53 and Bax, and upregulating the expression of antiapoptotic protein Bcl-2 [39]. Naguib reported a greater antioxidant activity in AST in comparison to different carotenoids, such as *α*-carotene, lycopene, lutein, and *ß*-carotene [40]. In vivo and in vitro studies on rats have shown that AST affects immunity; however, no clinical research has been conducted on humans [21, 41].

## 3. Ocular Diseases

### 3.1. Clinical Study

Preclinical and clinical evidence supports the potential use of AST in the prevention and treatment of a number of ocular diseases. The existing clinical research relating to AST primarily focuses on dry eye syndrome (DES), and age-related macular degeneration (ARMD) [14, 42, 43] (Table 1).

DES is a disorder of tear deficiency or excessive tear evaporation, resulting in an unstable tear film [46]. In a prospective, single-center, one-group, quasiexperimental study, a supplement of 6 mg AST twice a day improved the symptoms and signs of patients with mild-to-moderate DES [42]. The index, such as ocular surface disease index (OSDI) questionnaire score, noninvasive tear break-up time (NIBUT), fluorescein break-up time (FBUT), corneal fluorescein staining (CFS) score, eyelid margin signs, meibomian gland (MG) expressibility, meibum quality, and blink frequency improved significantly to varying degrees after treatment compared with those before the treatment [42]. Furthermore, a prospective, randomized, double-blinded study demonstrated that oral AST supplementation may increase tear production and improve tear film stability by reducing levels of ROS [14]. Further large and long-term studies are needed to determine the benefits of oral AST supplementation in patients with DES.

Age-related macular degeneration (ARMD) is the leading cause of visual impairment and blindness in some developing countries and developed countries among people aged over sixty-five years. ARMD is characterized by retinal pigment epithelium (RPE) cells within the damaged macula, leading to foveal photoreceptors loss [47]. AST intake leads to prevention and inhibition of dry ARMD progression [48]. A randomized controlled trial (RCT) reported that supplementation with carotenoids and antioxidants (including vitamin C (180 mg), lutein (10 mg), zeaxanthin (1 mg), and AST (4 mg) selectively improved central retinal dysfunctions (0°–5°). However, no functional changes were present in the more peripheral (5°–20°) retinal areas [43]. Another two-year RCT study demonstrates that oral lutein/zeaxanthin combined with additional carotenoids (AST) can clinically improve and stabilize visual acuity, contrast sensitivity, and visual function in ARMD patients [44]. However, carotenoid mixtures including AST were used in the above studies, and there has been no direct study that shows the effectiveness of AST clinically.

Three clinical studies were conducted to explore the mechanism of AST in protecting visual functions [1, 49, 50]. AST intake may have affected vascular endothelial growth factor (VEGF) levels in the aqueous humor through its antioxidant effects by increasing O_2_^.-^ scavenging activity and suppressing peroxide production [1]. Moreover, these previous studies reported that the effects of AST on VEGF level and antioxidative activities differed by sex. The VEGF level in the female aqueous humor was regulated by the overall extent of oxidation. Furthermore, AST affected O_2_^.-^ scavenging and participated in the regulation of VEGF level in the female aqueous humor [49]. Saito et al. confirmed that AST increased the choroidal blood flow velocity in the macular region, improved the local blood circulation in the eyes, and improved the ocular circulation. It was a randomized, double-blind, placebo-controlled trial where 20 normal subjects took 12 mg AST a day for four weeks [50]. This study has shown that AST significantly increased the choroidal blood flow with no side-effects; however, it is still unknown as to whether this effect is temporary or permanent [50].

### 3.2. In Vivo Animal Study

Numerous animal studies have further confirmed the protective effects of AST on the eye and its effectiveness in the prevention of ocular diseases, ranging from the ocular surface to the fundus, including DES, phototoxic keratitis [44], cataract [51], diabetic retinopathy [11], ARMD [48, 52], retinal ischemia injury [53], and other ocular diseases. All the details are presented in Table 2.

Under normal circumstances, the cornea absorbs most of the UV-B and UV-C radiation and protects the inner eye against UV-induced damaging effects. Exceeding the threshold levels of UV-B can induce inflammation of the cornea; this is called photokeratitis, which appears 6 to 12 hours after exposure [61]. The mechanisms involved include oxidative stress, DNA damage, and death receptor activation, all of which lead to apoptotic cell death [62]. Recently, Lennikov et al. reported that the topical administration of AST provided successful prevention against UV-induced photokeratitis in mice. AST protected the corneal epithelium in a dose-dependent manner by inhibiting ROS production and NF-*κ*B expression, further inhibiting inflammation. The anti-inflammatory activity of AST may be related to its antioxidant activity. In addition, they found that AST can suppress phototoxicity in the cultured mouse corneal epithelial cells (TKE2) in a dose-dependent manner. It prevents apoptosis of TKE2 cells by inhibiting NF-*κ*B activation and reducing oxidative stress. This suggests that AST might be a candidate treatment against the deleterious effects of UV exposure, with better clinical benefits as compared to other molecules [44]. Harada et al. produced similar results. AST protects corneal epithelium cells by decreasing the level of ROS and inhibiting NF-*κ*B activation and caspase-3-induced apoptosis. Inflammation was reduced by reducing the expression of cyclooxygenase-2 (COX-2), TNF-*α*, and CD45 in the corneal tissue [13]. Protective effects of AST on UV keratitis have not been proved in humans and further studies are needed.

Cataract is the leading cause of blindness and is an important cause of low vision globally [63]. The mechanism of cataract development is not clearly defined. It is a multifactorial disease, and oxidative stress might be the leading cause. Oxidative damage to lens proteins is a major factor in cataract formation [64]. Under the action of external harmful or deleterious factors (ultraviolet radiation, drugs, changes in self-metabolic state), excessive free radical production and impaired antioxidant defense system causes lens epithelial cell apoptosis, lens protein aggregation, increased ratio of insoluble protein to soluble protein, and impaired barrier function of the lens capsule [65]. These changes promote the occurrence and development of cataracts. Our study revealed that AST decreased the levels of glutathione (GSH), SOD, and CAT and increased the levels of malondialdehyde (MDA) and advanced glycation end products (AGEs) in the lens of rats with type I DM. AST also ameliorated the impaired morphologic changes and imparted a protective effect on diabetic cataracts [51]. The beneficial effect of AST on cataracts was also investigated in other experimental animal models. It was demonstrated that treatment with AST reduced lens opacity and prevented the development and progression of steroid-induced cataracts in chick embryo models. The researchers also showed that AST reduced glutathione levels in the lens [54]. It is recommended to do further research about the mechanism of AST as treatment for cataracts.

As mentioned above, the effect of AST on AMD has been widely studied in clinical research. The pathogenesis has also been further studied. An in vivo animal study considered the effect of AST on photoreceptor cell damage caused by light and pathological retinal angiogenesis. With age, cumulative photodamage and retinal susceptibility lead to ROS accumulation and a significant decrease in enzymes, such as SOD, CAT, and GPx [66]. Tomohiro et al. reported that AST protected the photoreceptor cells at a dose of 100 mg/kg and inhibited retinal dysfunction in terms of electroretinogram (ERG) and outer nuclear layer (ONL) loss and reduced the expression of apoptotic and 8-OHdG-positive cells, induced by light exposure via its antioxidative effect mechanism. Moreover, AST protected against increases of PI-positive cells and intracellular ROS activity in retinal core cells [48, 52]. Pathological neovascularization of the retina is another important cause of choroidal neovascularization (CNV) and AMD [57]. Otsuka et al. found the possibility of AST supplementation as a therapeutic strategy in suppressing CNV-associated ARMD in C57BL/6J mice [15]. AST can also downregulate the expression of VEGF, which can be used as a potential nutritional drug to prevent or treat retinal dysfunction in diabetic patients. AST can block the activation and translocation of HIF1*α* and XBP1 induced by high glucose or CoCl and downregulate the expression of HIF1*α* and XBP1. In addition, AST protected the disintegration of occlusion zone-1 (ZO-1) tight junction proteins in RPE and reduced the permeability of RPE cells induced by Mercury or hypoxia [11]. In addition, intravitreal and intraperitoneal AST administration can both inhibit retinal endothelial cell proliferation and exert an antiangiogenic effect. AST also offers a dose-independent reduction in mitochondrial injuries and a dose-dependent inhibition of apoptosis in a HIR newborn mouse model. The most effective route seems to be intravitreal high-dose AST injection [57]. In conclusion, AST can protect the RPE layer from oxidative damage and prevent CNV formation, thus, delaying the occurrence and development of ARMD. These results may provide great advancements in the treatment of such disease.

Diabetic retinopathy (DR) is one of the important and common microvascular complications of diabetes mellitus (DM) [67]. It is reported as one of the major causes of blindness and visual impairment in adults [68]. Oxidative stress and inflammation are the two determinant factors in the initiation and progression of DR [69]. High oxygen consumption makes the retina more vulnerable to oxidative stress. Thus, ROS induced apoptosis in retinal ganglion cells or photoreceptor cells plays an important role in DR. This increases the release of inflammatory cytokines thru the activation of NF-*κ*B [70, 71]. Yeh reported that AST significantly reduces the levels of oxidative stress mediators (8-OHdG) and inflammatory mediators, such as intercellular cell adhesion molecule-1 (ICAM-1), monocyte chemoattractant protein-1 (MCP-1), and fractalkine (FKN). Moreover, AST also increases the levels of antioxidant defense enzymes, such as HO-1, and inhibits NF-*κ*B activity, which promotes the release of inflammatory mediators, indicating that AST may be a beneficial nutritional supplement to halt the progression of DR and prevent vision loss in patients with diabetes in the retinas of streptozotocin-induced diabetic rats [56]. Similarly, Baccouche et al. found that short-term treatment of AST attenuated glial dysfunction and promoted the expression of oxidative stress marker HO-1, reducing glial reactivity in the diabetic retina of rats and exhibiting a protective effect [55]. However, the short-time administration of AST did not rescue the expression of the retinal ganglion cell marker, Brn3a, and synaptophysin, a marker of presynaptic vesicles [55]. AST improved oscillatory potentials (OPs) and decreased apoptosis of retinal ganglion cells (RGCs) and inhibited the increase of MDA and 8-OHdG levels; it showed an antioxidative effect in the retina of db/db mice [16].

Retinal ischemia is a common clinical condition that often leads to visual impairment and blindness [72]. Ischemia in the retina and optic nerve is involved in the pathogenesis of major retinal diseases, such as ARMD, DR, and glaucoma. Ischemia induces the release of neurotransmitters, which causes membrane depolarization, increase in intracellular calcium concentration, and production of nitric oxide (NO) and ROS [73, 74]. When ROS, such as superoxide anion radicals, their dismutation product H2O2, and hydroxyl radicals are overproduced, they induce cell death [75]. In the present study, oral administration of AST at 100 mg/kg significantly prevented ischemic retinal cell damage [76]. Otsuka et al. demonstrated the protective effects of AST against retinal histological damage after ischemia or reperfusion injury. AST increases the number of RGC and the thickness of inner plexiform layer (IPL) and inner nuclear layer (INL) by reducing ROS production [53]. Oral administration of AST might be effective in protecting against neurodegeneration during ischemic retinopathy.

Glaucoma is a chronic neurodegenerative disease characterized by high intraocular pressure, which is the main cause of blindness worldwide [77]. It can lead to apoptosis of RGCs and gradual loss of optic nerve axons, further causing visual field defects in glaucoma patients [78]. AST reduced retinal oxidative stress injury by activating the Nrf2/HO-1 pathway, thereby inhibiting RGC apoptosis and protecting the integrity of the retinal structure. Moreover, Li et al. reported that AST can significantly alleviate the symptoms of patients during adjuvant treatment for acute glaucoma [79]. Cort et al. also found that AST had a protective effect on the retinas in rats with elevated intraocular pressure (EIOP). The amplitude of visual evoked potential (VEP) P100 in the experimental group returned to control levels after AST treatment. AST had an inhibitory effect on apoptosis of RGCs, demonstrating the protective effect of AST in glaucoma and other optic neuropathies [12]. AST exerts antioxidative stress and neuroprotective effects on inhibiting RGC degeneration and retinal degeneration in a mouse model of NTG, indicating that AST may be a potential therapeutic agent for the treatment of glaucoma [59].

Few studies have assessed retinal drug toxicity induced by antitumor drugs and uveitis [60, 80]. Cisplatin (CIS) is widely used to treat various cancer types, such as head, neck, ovarian, testicular, breast, and small lung cell [80–82]. However, as a rare condition, its retinal toxicity is stated in a few case reports and its mechanism is still unclear [60, 81]. Fındık et al. found that AST treatment reduced the increase in MDA, eNOS, and 8-OHdG levels and increased the levels of GSH expressions in the retinas of rats following CIS administration [60]. However, the antitumor effect of CIS was explained by DNA adduct formation, ROS accumulation, lipid peroxidation, and mitochondrial stress increase. Hence, further studies are necessary to define the optimal doses of AST for protecting the retinal tissues without sacrificing the main purpose of CIS therapy in the future [60]. With regard to the uvea, there are only a few studies on the effect of AST on uveitis. Studies have shown that AST can significantly decrease the number of infiltrating cells in the anterior chamber and the concentration of protein, NO, TNF-*α*, and prostaglandin E2 (PGE2) in the aqueous humor in rats with endotoxin-induced uveitis (EIU). The number of activated NF-*κ*B-positive cells was lower in the iris-ciliary bodies, which suggests that AST reduces ocular inflammation in the eyes with EIU by downregulating proinflammatory factors and by inhibiting the NF-*κ*B-dependent signaling pathway [80]. AST also has an anti-inflammatory effect on the choroid.

### 3.3. In Vitro Study

In studies that investigated the effect of AST on ocular diseases, the common cell models included RPE cells [15], photoreceptor cells [83], retinal ganglion cells(RGCs) [84], corneal epithelium cells [85], and lens epithelial cells [86], which correspond to one or more ocular diseases and elaborate relevant mechanisms (Table 3). The effect of AST on RPE is currently the most researched in in vitro studies. Li et al. found that AST clearly reduced H2O2-induced ARPE-19 cell viability loss, cell apoptosis, and intracellular generation of ROS via Nrf2-mediated upregulation of the expression of phase II enzymes, involving the PI3K/Akt pathway [88]. In addition, Izumi et al. found that AST could inhibit the expression of inflammation-related molecules and angiogenic molecules, including VEGF, IL-6, ICAM-1, MCP-1, and vascular endothelial growth factor (VEGFR-1 and VEGFR-2). Furthermore, AST suppressed the activation of the NF-*κ*B pathway, including NF-*κ*B degradation and p65 nuclear translocation. They also suggested that AST had an early intervention effect on CNV [15].

AST mainly exerts antioxidative and antiapoptotic functions in photoreceptor cells. Recent studies have reported that AST can protect against light-induced injury of the photoreceptor cells by activating nuclear factor 2 (Nrf-2) and by reducing ROS production [52]. Nrf2 is a transcription factor that regulates the antioxidant responses via modulating the expression of phase II enzymes expression such as heme oxygenase-1 (HO-1), NAD (P) H: quinone oxidoreductase (NQO-1), and g-glutamyl-cysteine ligase (GCL) [83]. Lin et al. reached a similar conclusion that AST inhibited the blue light-emitting diode-induced apoptosis, ROS production, expression of oxidative stress biomarkers (8-OHdG, nitrotyrosine, and acrolein), and mitochondrial damage in an LED-induced retinal injury cell model by free radical scavenging activity, inducing phase II antioxidant enzyme expression, and activating the PI3K/Akt/Nrf2 pathway [83]. Light can also cause corneal epithelial damage, and AST has a certain protective effect on the lens due to its strong antioxidant capacity, preventing UVB-induced lipid peroxidation and stress signaling (JNK and p38 activation) in human lens epithelial cells [86].

The antioxidant effect of AST also imparts as a neuroprotective effect against glutamate-induced RGC apoptosis [85]. In a previous study, AST protected the RGCs from apoptosis and damage via decreasing ROS production. The retinal oxidative DNA damage and lipid peroxidation damage were also mitigated [58]. Yamagishi et al. reported that AST reduced the ROS production. Moreover, the generation of the DNA damage marker, 8-OHdG, could inhibit apoptotic and RGC necrosis caused by glutamate, hypoxia, and oxidative stress [84].

Several studies have shown that AST prevents DES through antioxidative and anti-inflammatory effects [88]. Chronic inflammation and oxidative stress have been shown to play a role in DES [89, 90]. Free radicals attack the cellular plasma membrane and can cause corneal epithelial cell damage and death. They also damage the epithelial tissues of the conjunctiva, the lacrimal glands, and tear secreting tissues [91]. Antioxidants protect against free radicals and provide a stable tear film [92]. Furthermore, reports on AST treatment have shown that it prevents aging-related hyposalivation due to dysfunction of the submandibular glands by decreasing inflammatory cells and increasing aquaporin-5 positive cells. Shimokawa et al. confirmed that upregulation of age-related markers, such as p53 and p21, increases intracellular ROS and oxidative stress, thereby leading to the development of DES. AST not only directly inhibited the generation of ROS in corneal epithelium cells in an in vitro model of dry eye syndrome, but also decreased the level of age-related markers, such as p53, p21, and p16, which were known to induce the generation of intracellular ROS and apoptosis of human corneal epithelial cells. Their results demonstrated that high affinity liposomal AST could be a potential candidate for the treatment of dry eyes [85].

## 4. Conclusion

**Figure 1:**
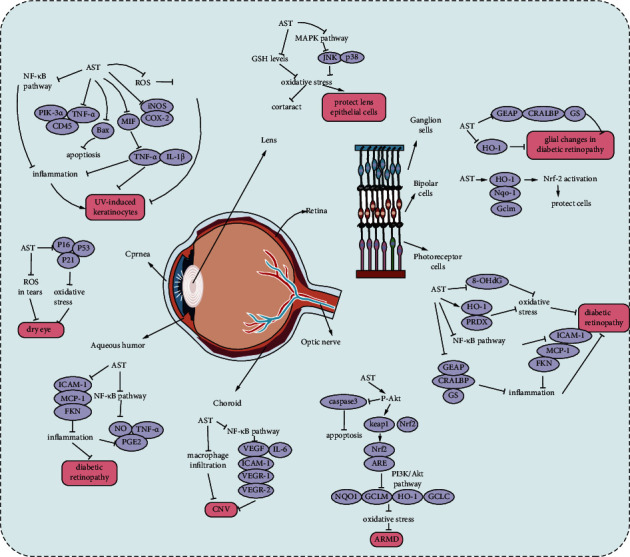
An illustration of AST targets in the ocular diseases.

AST, as a natural extract with strong antioxidative activity, can delay the progress of many diseases and protect multiple organs of the whole body. Toxicological analysis showed that the compound had good safety and bioavailability [93]. Although not officially approved by EFSA or FDA, AST has tremendous potential in the global health product market. AST is a promising ophthalmic drug. Most ophthalmic diseases, such as dry eye syndrome, keratitis, cataract, and age-related macular degeneration, are associated with oxidative stress and inflammation that lead to the production of reactive intermediates and cell apoptosis. In addition, ultraviolet radiation, chemotherapy, and other processes can induce retinal cell apoptosis. In this article, we reviewed the protective mechanism of AST in ophthalmic diseases (Figure 1). Further in-depth studies employing animal models or cell lines will be beneficial.

## Figures and Tables

**Table 1:** Summary of in clinical studies on eye and AST.

Study type	Number	AST dose and duration	Referred ocular disease	Biological effects	Index	References
Prospective,	60 patients/120 eyes	12 mg/day	DES	Improve the symptoms and signs	Osd, tbut, FBUT, CFS, MG	[42]
One-group		30 ± 2 days				
RCT	43	20 mg/kg/day	DES	Improve tear production and tear film stability	Schirmer test, TBUT	[14]
		8 weeks		Reduce levels of ROS		
RCT	20	12 mg/day	ARMD	Choroidal blood flow velocity	—	[44]
		4 weeks				
RCT	27	4 mg/day	ARMD	—	mfERG	[43]
		12 months				
RCT	145	4 mg/day	ARMD	Stable/improve contrast sensitivity and visual acuity	—	[45]
		12 months				
RCT	35	6 mg/day 2 weeks	ARMD	Affect O2.- scavenging activity in the aqueous humor	—	[1, 49]

**Table 2:** Summary of in vivo animal studies on eye and AST.

Study type	Animals	AST dose	Referred ocular disease	Biological effects	Related genes	References
In vivo	C57BL/6J mice	1/0.1/0.01 ng/ml by eye drop	Phototoxic keratitis	Increase corneal thickness, antiapoptosis, reduce ROS and NF-*κ*B	NF-*κ*B	[44]
In vivo	C57BL/6J mice	6mg	Phototoxic keratitis	Improve corneal epithelial thickness	NF-*κ*B , caspase-3, CD45, COX-2, TNF-*α*,I*κ*B	[13]
				Antiapoptosis		
				Inhibit activity of NF-*κ*B		
				Decrease proinflammatory factors and ROS		
In vivo	Chicken embryo model	50/80/100 mg/ml	Cataract	Inhibit HC-induced cataract	—	[54]
				Reduced GSH levels		
In vivo	Sprague Dawley rat	16/80 mg/kg	Cataract	Delay progress of cataract	AGEs, SOD, GSH, CAT, MDA	[51]
				Protect epithelium of lens		
				Decrease antioxidant levels		
In vivo	Db/db rats	25/5 mg/kg by oral gavage	Type 2 diabetes mellitus	Decrease levels of oxidative stress marker	MDA, 8-OHdG, SOD	[16]
				Inhibit apoptosis		
In vivo	*Psammomys obesus*	10 mg/kg	Type 2 diabetes mellitus	Increase HO-1 expression	HO-1, glial markers (GFAP, CRALBP, and GS)	[55]
				Attenuates glial dysfunction		
In vivo	Wistar rats	0.6/3 mg/kg	Type 1 diabetes mellitus	Decrease oxidative stress and inflammatory mediators and inhibit activity of NF-*κ*B	8-OHdG, NO, acrolein, ICAM-1, MCP-1	[56]
				Increase antioxidant enzyme		
In vivo	ddY mice	100 mg/kg	Retina ischemia (AMD, DR, glaucoma	Protect retinal functional and histological damage	—	[53]
				Decrease RGCs death		
In vivo	C57BL/6J mice	10/100 ng/*μ*L	Pathological retinal angiogenesis	Antiproliferation and antiapoptosis of endothelial cells	—	[57]
In vivo	C57BL/6J mice	1/10/100 mg/kg	AMD	Suppress CNV and macrophage infiltration	ICAM-1, MCP-1, IL-6, VEGF, VEGFR-1, VEGFR-2, I*κ*B	[15]
				Decrease expression of inflammatory and angiogenic molecules		
				Inhibit activity of NF-*κ*B		
In vivo	ddY mice	100 mg/kg	AMD/RP	Increase GCL and INL	8-OHdG, 4-HNE	[58]
				Inhibit oxidative DNA damage and lipid peroxidation damage		
				Antiapoptosis		
In vivo	ddY mice	100 mg/kg	AMD/RP	Ameliorate retinal dysfunction and histological damage	8-OHdG, ROS	[48]
				Antiapoptosis		
In vivo	Glast ± mice and C57BL/6J mice	10/30/60 mg/kg	Normal intraocular glaucoma (NTG)	Suppress RGCs loss	4-HNE, pI*κ*B, I*κ*B	[59]
In vivo	C57BL/6J mice	50 mg/kg	Glaucoma	Protect retinal tissues and RGCs from oxidative stress	Bax, Bcl-2, Nrf2. HO-1, ROS	[11]
				Inhibit apoptosis of RGCs		
				Active nrf2/HO-1 pathway		
In vivo	Sprague Dawley rat	25/75 mg/kg	Retinal toxicity	Prevent retinal toxicity by CIS	GSH, MDA	[60]

**Table 3:** Summary of in vitro studies on eye and AST.

Study type	Cell lines	AST dose	Referred ocular disease	Biological effects	Related genes	References
In vitre	ARPE-19	0/5/10/20 *μ*m	AMD	Attenuated H2O2-induced oxidative stress	NQO1, HO-1, GCLC, GCLM, Nrf2, PI3K, Akt, caspase 3	[87]
				Active Nrf2-ARE, PI3K/Akt pathway		
In vitro	ARPE-19	50/150 *μ*m	AMD	Inhibit inflammatory and angiogenic molecules and NF-*κ*B activation	IL-6, VEGR-1, VEGR-2, MCP-1, ICAM-2, I*κ*B, NF-*κ*B, p65	[15]
In vitro	661W cells	3/10/20 *μ*m	AMD	Active Nrf2	Nrf2, c-Jun, phaseII enzymes (HO-1, Mqo-1, GCLM)	[52]
				Reduce ROS production		
				Mitigate photoreceptor cell death		
In vitro	661W cells	0–50 *μ*m	AMD	Reduce ROS production	Bcl-2, Bax, PI3K, Akt, ROS phaseII enzymes (HO-1, NQO1)	[83]
				Inhibit cell death, oxidative stress markers, phaseII enzymes expression and Nrf2/PI3K/Akt pathway		
In vitro	RGC-5	0.01/0.1/1/10 *μ*m	Glaucoma	Reduce cell death and ROS production	—	[58]
In vitro	RGC-5	1/10/100 nm	Glaucoma	Increase survival rate of cell death induced by glutamate/oxidative stress/hypoxia	—	[84]
				Inhibit DNA oxidative damage, apoptosis and necrotic RGCs death		
In vitro	RGC-5	0.1–10 *μ*m	Retinal ischemia reperfusion	Reduced ROS production	—	[53]
In vitro	HCECs	0.2/1/2/10 *μ*m	Phototoxic keratitis	Increase cell viability	ROS, p53, p32, p16	[85]
				Reduced ROS production and age-related factors		
In vitro	HCECs TKE2 cells	1/0.1/0.01 mg/ml	Phototoxic keratitis	Decrease cytotoxicity	8-OHdG	[44]
In vitro	HLECs SRA 01–04	1/2/4 *μ*m	cataract	Inhibit UV-induced oxidative stress activity and lipid peroxidation	p-JNK, p38	[86]
